# Editorial: Cell-Cell and Cell-Matrix Adhesion in Immunobiology and Cancer

**DOI:** 10.3389/fimmu.2019.03126

**Published:** 2020-01-23

**Authors:** Toshiyuki Murai, Hiroto Kawashima, David Naor

**Affiliations:** ^1^Department of Microbiology and Immunology, Graduate School of Medicine, Osaka University, Suita, Japan; ^2^Laboratory of Microbiology and Immunology, Graduate School of Pharmaceutical Sciences, Chiba University, Chiba, Japan; ^3^Faculty of Medicine, Lautenberg Center of Immunology, Institute for Medical Research Israel-Canada (IMRIC), Hebrew University of Jerusalem, Jerusalem, Israel

**Keywords:** cell adhesion molecule, extracellular matrix, immune system, cancer progression, cytoskeleton, cell dynamics

Cell-cell interactions and cell-extracellular matrix (ECM) interactions guide complex cellular decisions in various physiological processes including immune regulation such as leukocyte trafficking via blood and lymphatic vascular system. The interactions are also critical for the development of diseases and disorders including immune disorders, cancers, cardiovascular diseases, and neurodegenerative diseases. These interactions are mediated at the cell surface by adhesion molecules including integrins, selectins, CD44, and lectin molecules such as sialic acid-binding immunoglobulin-like lectins (Siglecs) as well as cell-cell interaction molecules such as cadherins. In addition, these interactions are mediated by ECM molecules such as hyaluronan and proteoglycans. Recent experimental evidence has indicated that these processes are finely tuned by the supramolecular assembly of adhesion molecules and cytoskeletal proteins that sense and recognize the physical properties of environmental cues. In light of the current need for new approach to treating and preventing diseases such as immune disorders and cancer and due to the complex nature of the molecular sociology of the cell, it has been increasingly evident that a multidisciplinary approach to evaluate them is crucial. We invited leading international researchers in the field to this Research Topic, which comprises seven reviews and two original articles and provides a timely and comprehensive view of recent advances in the understanding of cell-cell and cell-ECM interactions in the regulation of immune cell function and cancer particularly for potential cancer immunotherapy.

Cancer immunotherapy has shown great progress in the past decade, particularly focusing on immune modulation by immune checkpoint blockade (ICB), which has revolutionized cancer treatment and has fundamentally changed the outcome for certain groups of patients with advanced cancer. The 2018 Nobel Prize in Physiology or Medicine was awarded to Tasuku Honjo and James P. Allison, for their work on unleashing the body's immune system to attack cancer by targeting immune checkpoint molecules PD-1 (1) and CTLA-4 (2), respectively. Current attempts include the introduction of combination therapies of such novel therapeutic strategies with the conventional therapies, including chemotherapy. Si et al. performed a meta-analysis involving clinical trials with cancer patients and showed that incidence of peripheral neuropathy related to PD-1/PD-L1 inhibitors was significantly lower compared with chemotherapy group, while the risk was increased when it was combined with chemotherapy. On the other hand, at present, several trials to combine ICB with other emerging immunotherapies including adoptive cell therapy are exploring to further improve the outcomes.

Chimeric antigen receptor (CAR)–modified T cell therapy is also the emerging therapy to cancer. However, while CAR-T therapy has had success in the treatment of hematological tumors, it is much harder for CAR-T cells to attack solid tumors (3). One of the most important adhesion molecules for T cell homing is L-selectin, and Watson et al. reported that increasing L-selectin on anti-cancer T-cells improved immune control of solid tumor progression. This study revealed a novel role for L-selectin in cancer immunotherapy for solid tumors and offers great promise to serve as a novel methodology to enhance the efficacy of CAR-T therapy for solid tumors. The review by Ivetic et al. provides a detailed review for the biochemical properties of L-selectin in immune cell function and its diverse functions in leukocyte-endothelial interactions. Other lectins such as Siglecs also regulate immune responses by binding to cancer cell-derived glycans, yet the potential to target these molecules' interactions are currently poorly investigated. Läubli and Borsig describes the role of Siglecs in immune suppression in cancer with an original view that highlights three families of cell-cell interaction molecules: selectins, Siglecs and integrins that support cancer development. Authors further discuss the mechanisms during immune evasion and metastasis that can be therapeutically targeted by blocking these cell-cell interactions.

Dendritic cells (DCs) are key regulators of tumor immunity and thus DC cancer immunotherapy is another emerging method (4). Particularly, integrins play a pivotal role in various aspects of immune cell function by transmitting signals bidirectionally. Harjunpää et al. put forward an updated perspective of current knowledge on integrin function in immune cells including DCs and its relevance in cancer, and provide lists on anti-cancer clinical trials targeting integrins which have published results on clinical efficacy. Integrins are known to serve as mechanoreceptors for outside-in signaling through two transcriptional activators, yes-associated protein (YAP) and transcriptional co-activator with PDZ-binding motif (TAZ) operating in the Hippo pathway. An original article by Guenther et al. describes a novel mechanosensing link between β2 integrin and myocardin related transcription factor/serum response factor (MRTF/SRF) pathway that regulates DC migration and adhesion by using emerging techniques of three-dimensional cultures and quantitative imaging and physical measurement of traction force during cell adhesion.

Bidirectional communication between cancer cells and their tumor microenvironment (TME) is critical for cancer progression, and thus cancer cell-ECM interaction in TME may be a new target for immunotherapy (5). ECM is composed of two classes of macromolecules: (1) glycosaminoglycans and proteoglycans, and (2) fibrous proteins including collagens and laminins, among which the glycosaminoglycan hyaluronan has an impactful role in TME. Bourguignon focused on the role of hyaluronan and its receptor CD44 on RNA function, particularly on the function of micro-RNAs and long non-coding RNAs (lncRNAs). Liu et al. overview the complexity of hyaluronan and its interactions with receptors in TME in promoting or inhibiting cancers. Tzanakakis et al. presented a review on the role of proteoglycans including versican, syndecan and endocan in cancer development focusing on possible targets for cancer therapy. Classically, the generation of an efficient adaptive immune response against cancer occurs in secondary lymphoid organs (SLOs), while recent studies on TME revealed that they also occur directly at the tumor site called ternary lymphoid structures (TLSs) where lymphoid organs ectopically induced (6). Since evidence suggests that TLSs use a similar set of adhesion molecules to SLOs, the information on cell-cell and cell-matrix adhesion collected in this issue might be useful in understanding TLS-mediated anti-cancer immune responses and for modulating therapeutic response to ICB.

We wish that this collection will provide in-depth insights into current research on cell-cell and cell-matrix adhesion in immunobiology and cancer, and serve to discover and develop novel therapeutic approaches and the methods for prevention of diseases in the near future.

## Author Contributions

All authors listed have made a substantial, direct and intellectual contribution to the work, and approved it for publication.

### Conflict of Interest

The authors declare that the research was conducted in the absence of any commercial or financial relationships that could be construed as a potential conflict of interest.
